# Retraction Note: Neuron tau-targeting biomimetic nanoparticles for curcumin delivery to delay progression of Alzheimer’s disease

**DOI:** 10.1186/s12951-023-02077-w

**Published:** 2023-08-25

**Authors:** Chunhong Gao, Xiaoyang Chu, Wei Gong, Jinpeng Zheng, Xiangyang Xie, Yuli Wang, Meiyan Yang, Zhiping Li, Chunsheng Gao, Yang Yang

**Affiliations:** 1 State Key Laboratory of Toxicology and Medical Countermeasures, Beijing Institute of Pharmacology and Toxicology, Beijing, 100850 China; 2 The Fifth Medical Center of Chinese, PLA General Hospital, Beijing, 100071 China; 3 General Hospital of Central Theater of the PLA, Wuhan, 430070 China

**Retraction Note: Journal of Nanobiotechnology (2020) 18:71**

10.1186/s12951-020-00626-1

The Editors-in-Chief have retracted this article. After publication, concerns were raised regarding the images presented in the figures. Specifically:

Figure 8a western blots appear to have vertical breaks in the backgrounds.

Figure 12 Liver CUR and RPCNP-CUR images contain a highly similar area (rotated).

The authors have stated that some lanes have been removed from the western blots to exclude irrelevant samples, and that an incorrect image was used in Fig. 12 by mistake. However, the authors have been unable to provide the underlying raw data upon request. The Editors-in-Chief therefore no longer have confidence in the presented data.

Chunhong Gao and Yang Yang have not explicitly stated whether they agree to this retraction. None of the other authors have responded to any correspondence from the publisher about this retraction.

